# Supplementary material for: Ultra-lightweight rechargeable battery with enhanced gravimetric energy densities >750 Wh kg−1 in lithium–sulfur pouch cell
Source: Commun Eng. 2024 Nov 25;3:177. doi: 10.1038/s44172-024-00321-1 (PMC11589146; doi:10.1038/s44172-024-00321-1)
Supplement: Supplementary file 2 — Supplementary Information [file 44172_2024_321_MOESM2_ESM.pdf]

Supplementary Information

Ultra-lightweight rechargeable battery with enhanced gravimetric energy densities >750 Wh kg^{-1} in lithium–sulfur pouch cell

Kenji Kakiage[1]*, Toru Yano[1], Hiroki Uehara[2] and Masaki Kakiage[2]*

[1] Environmental & Energy Materials Laboratory, ADEKA CORPORATION, 7-2-35 Higashi-ogu, Arakawa-ku, Tokyo 116-8554, Japan

[2] Division of Molecular Science, Graduate School of Science and Technology, Gunma University, 1-5-1 Tenjin-cho, Kiryu, Gunma 376-8515, Japan

*Corresponding authors, E-mail addresses: ke-kakiage@adeka.co.jp (K. Kakiage), kakiage@gunma-u.ac.jp (M. Kakiage)

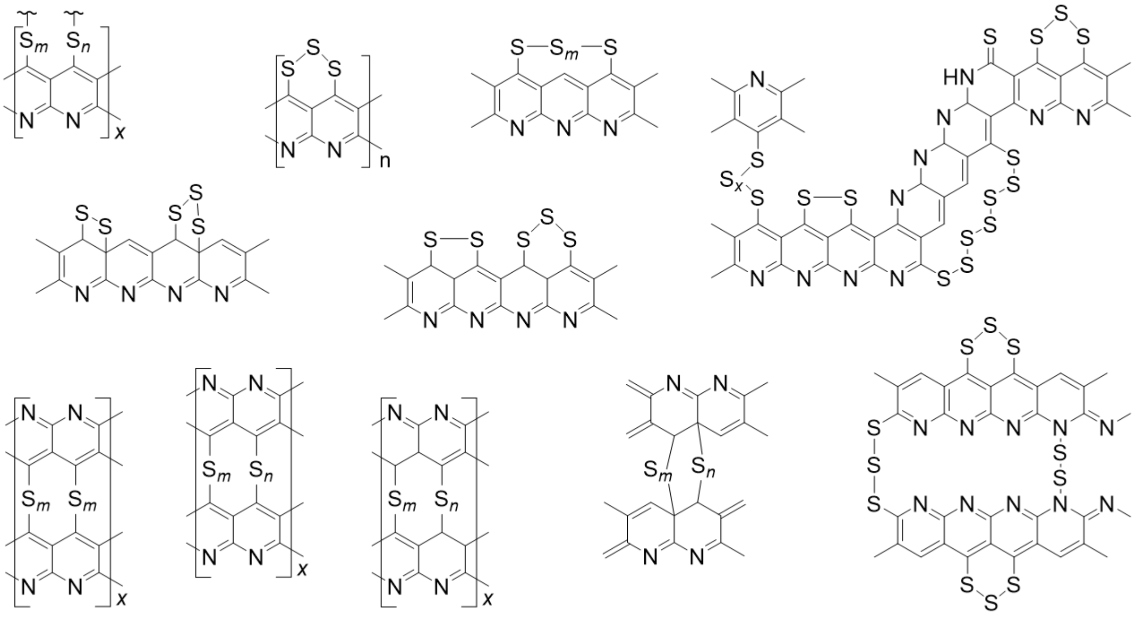

Supplementary Fig. 1 | Reported partial structures of SPANs before chg./dischg. operations[1–3].

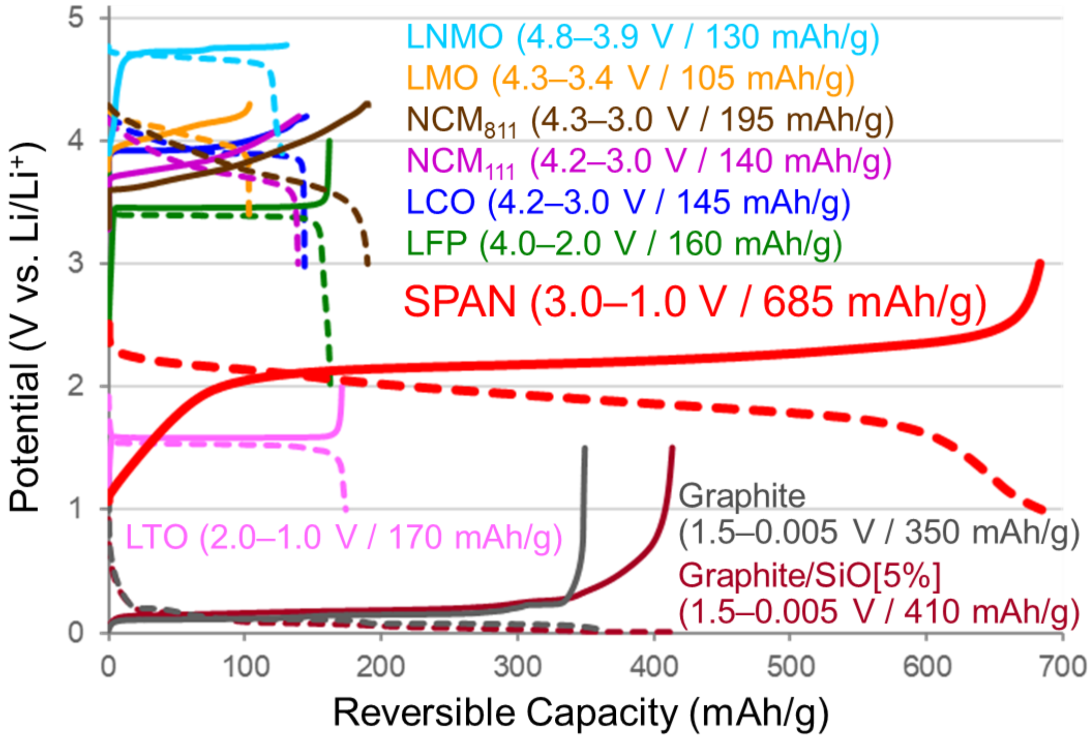

Supplementary Fig. 2 | Reversible chg./dischg. properties at 0.2C-rate and 30 ºC for various electrode active materials in coin cells (active materials | carbonate electrolyte solution | Li–metal anode).

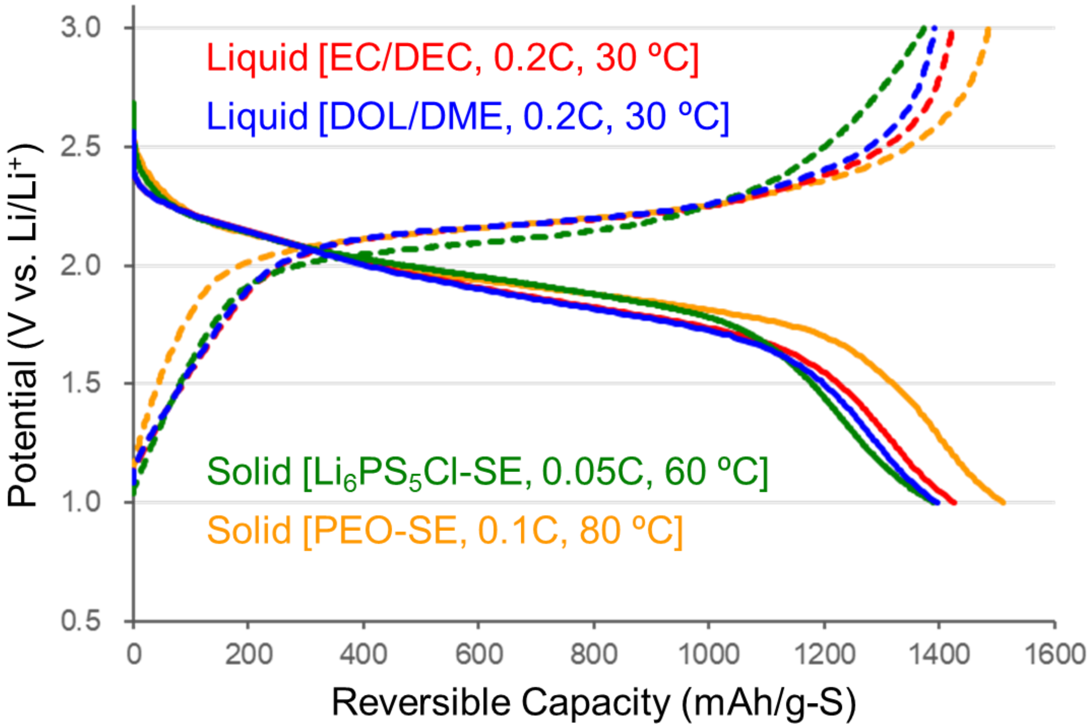

Supplementary Fig. 3 | Reversible chg./dischg. properties of SPAN cathodes with various liquid (carbonate and ether) or solid (sulfide-argyrodite and polymer) electrolytes.

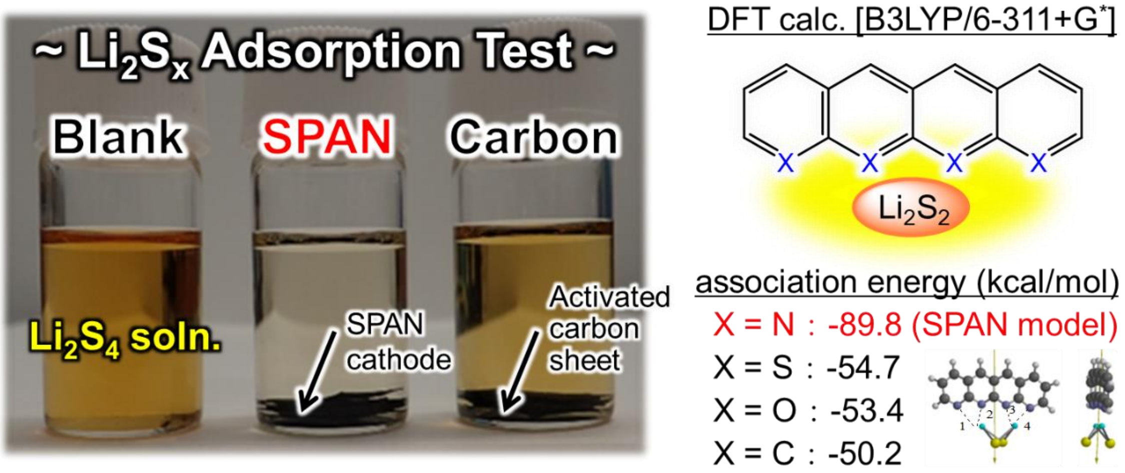

Supplementary Fig. 4 | Investigations of interactions between SPAN and lithium polysulfides (Li_2S_x): Visual adsorption ability tests using a Li_2S_4 solution[4,5] and association energy calculations between SPAN's backbone-model molecules and Li_2S_2 with the Gaussian 09 program package by using a density functional theory (DFT)[6]. A Becke's three parameter hybrid functional with the LYP correlation functional (B3LYP) was employed together with 6-311+G* basis set[7,8].

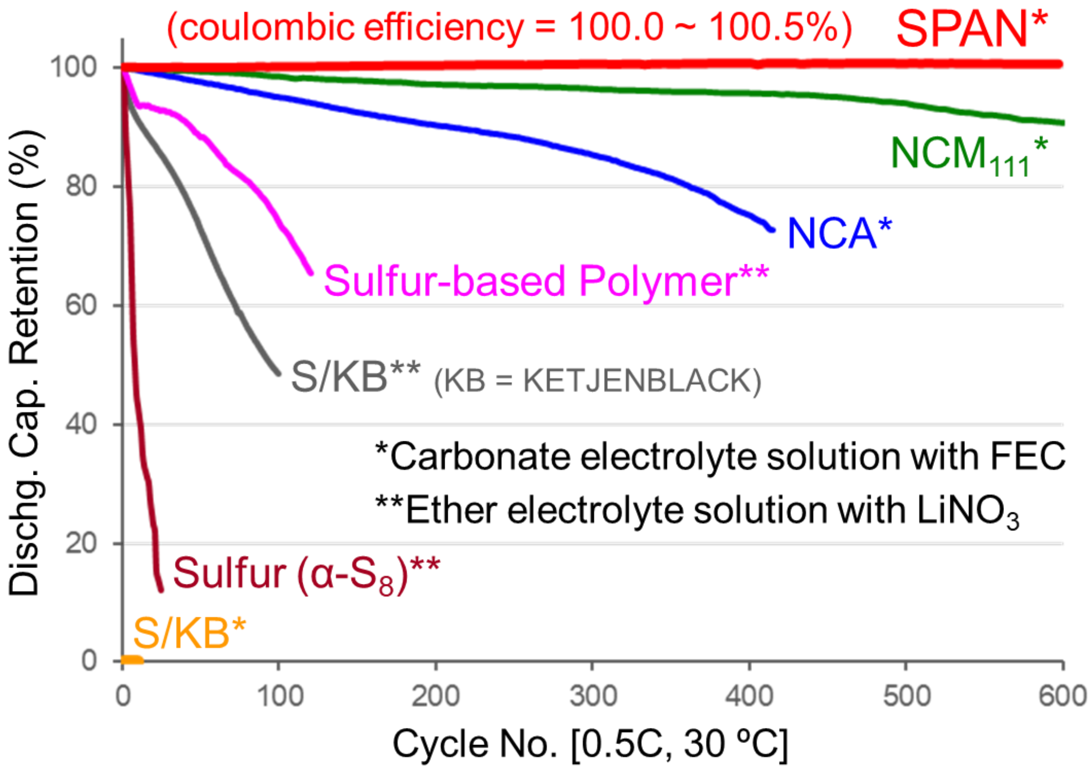

Supplementary Fig. 5 | Chg./dischg. cycle performances at 0.5C-rate and 30 ºC after a formation process of ten cycles at 0.1C-rate and 30 ºC for various cathode active materials in coin cells (active materials | carbonate or ether electrolyte solutions | Li–metal anode).

(a) (b)

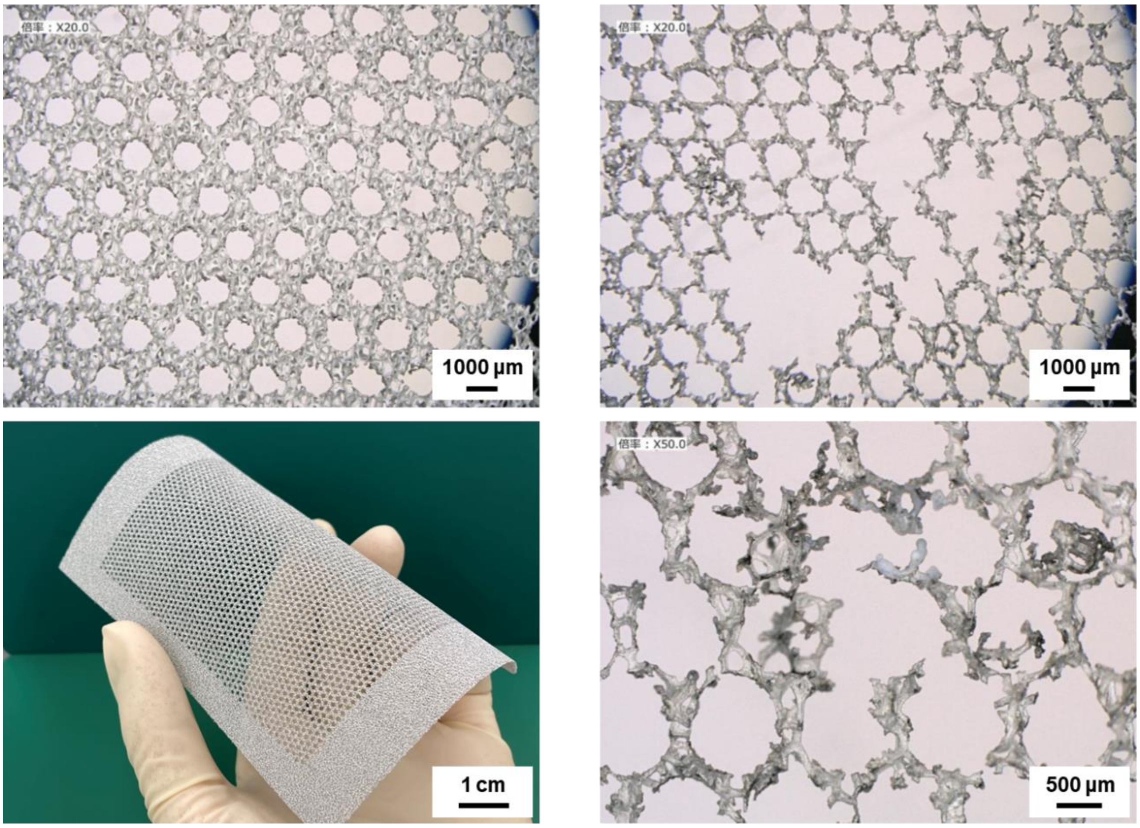

Supplementary Fig. 6 | Photographs of 3D-Al foam sheets (Al-CELMETs) weight-saved by the WIRED’s laser-drilling technique. **a**, Aperture area of 31% and flexibility. **b**, Aperture area of 46% and rupture points.

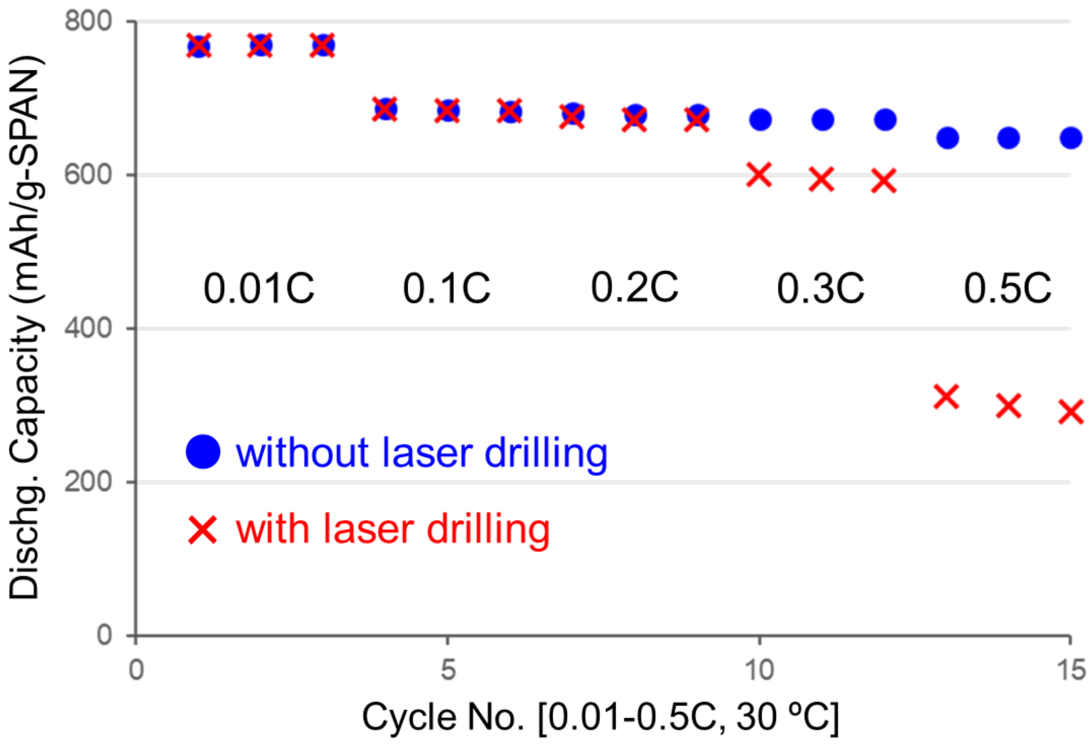

Supplementary Fig. 7 | Chg./dischg. cycle performances at 0.01–0.5C-rates and 30 ºC for SPAN cathodes in the 3D-Al foams with/without the laser drilling (31% aperture area) in pouch cells (SPAN cathodes | carbonate electrolyte solution | Li–metal anode).

(a)

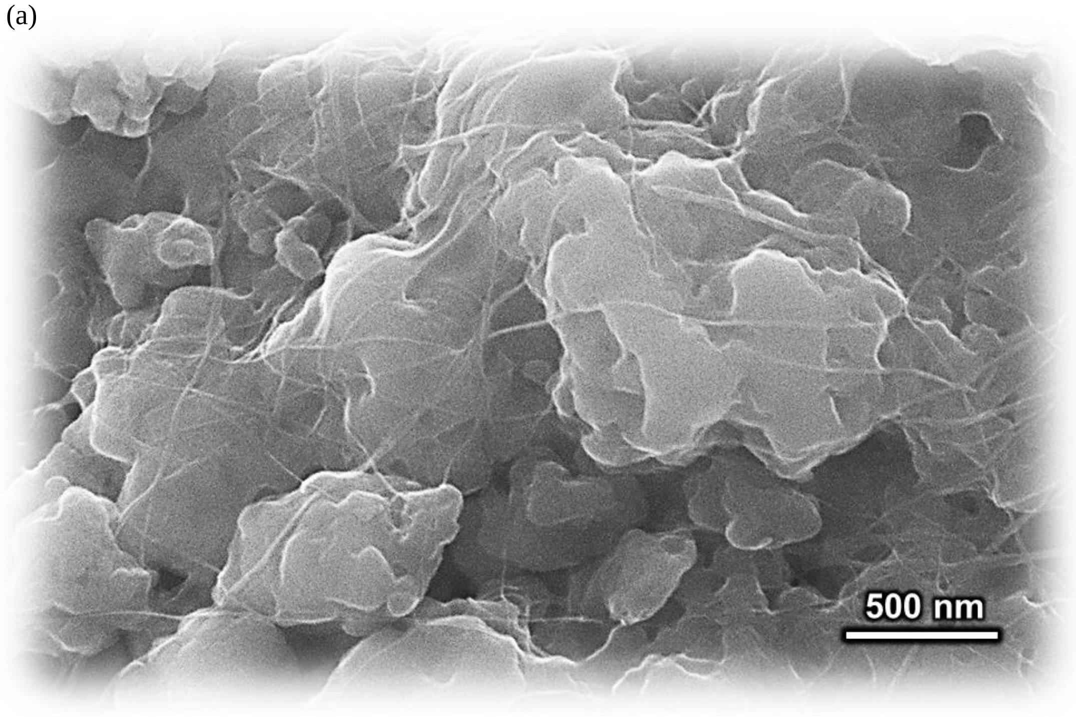

(b)

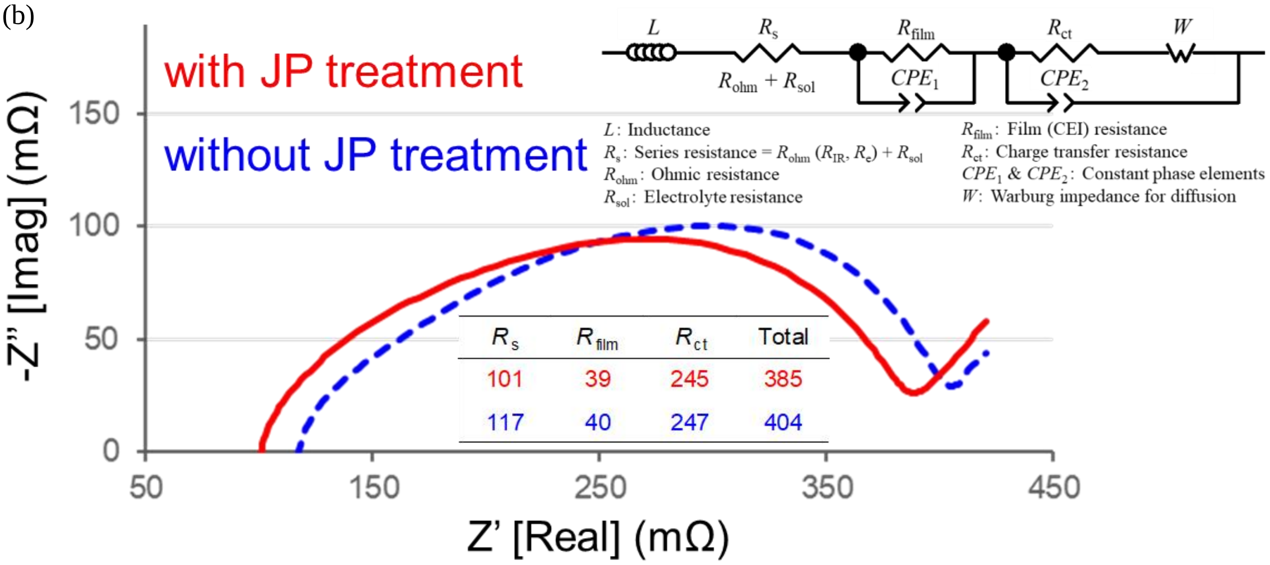

Supplementary Fig. 8 | a, SEM image of a SPAN cathode with the SWCNT as the conductive agent. **b**, Nyquist plots at SOC = 50% and 30 ºC of SPAN cathodes using SWCNTs with/without the Nihon Spindle Manufacturing's JET PASTER (JP) treatment in pouch cells (SPAN cathodes | carbonate electrolyte solution | Li–metal anode).

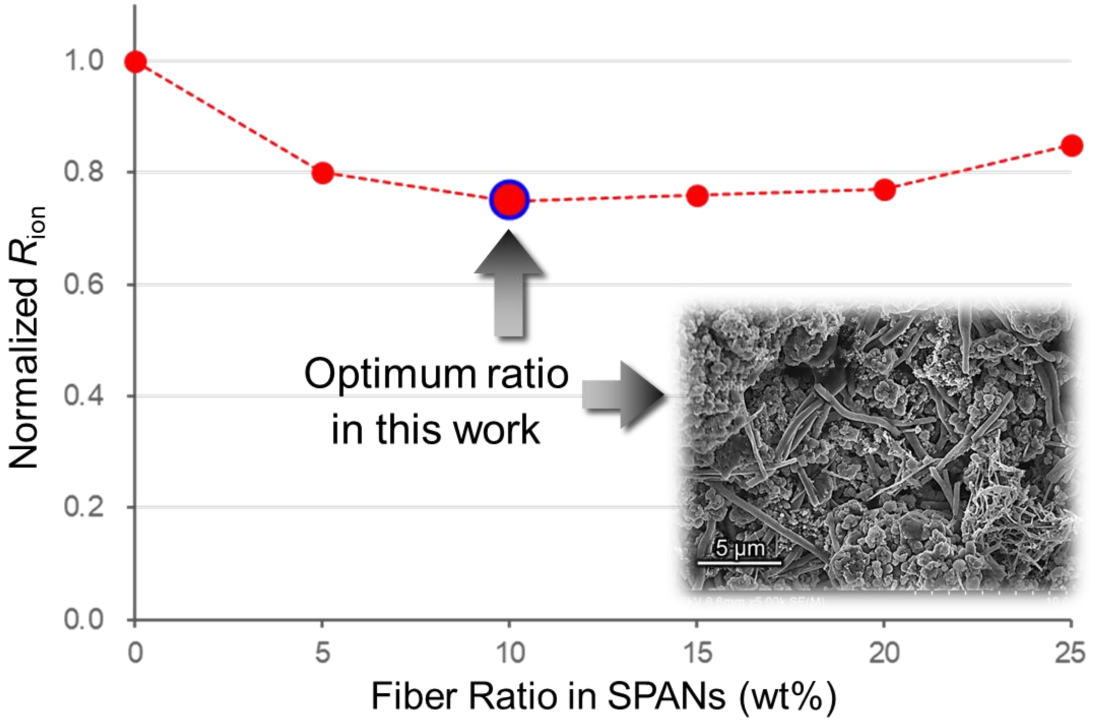

Supplementary Fig. 9 | Relationship between ion diffusion resistance (R_{ion}) estimated from EIS and fiber ratio in SPAN active materials with particle and fiber shapes.

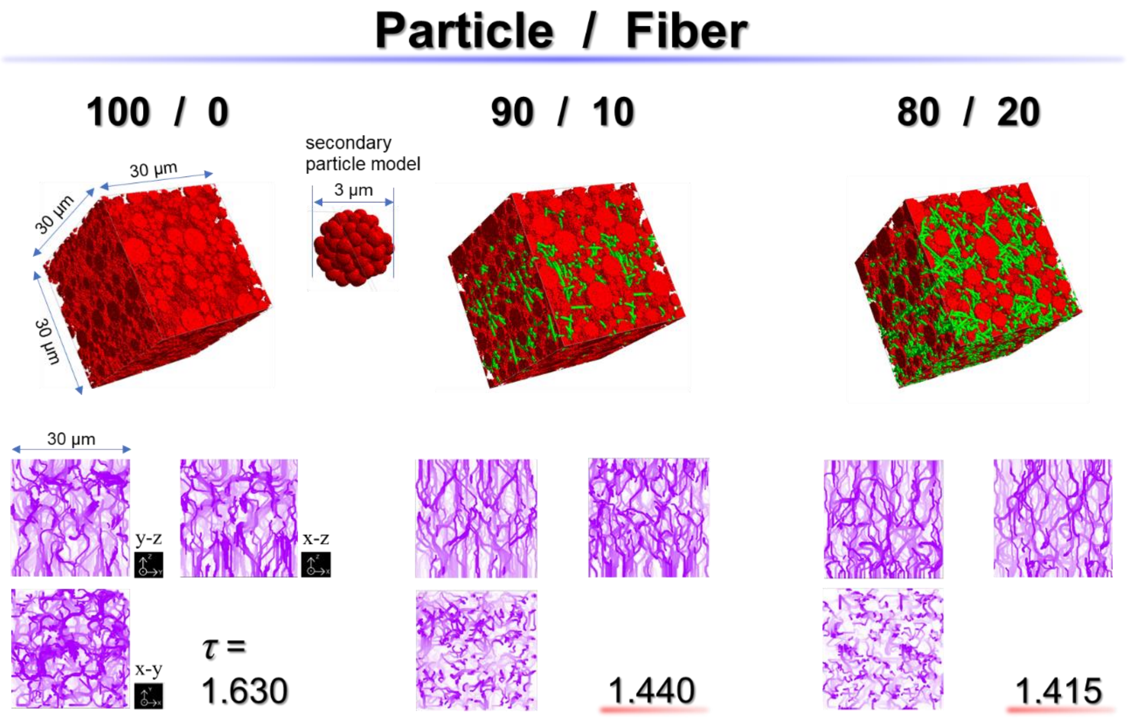

Supplementary Fig. 10 | GeoDict simulations of model SPAN cathodes consisting of secondary particles with D_{50} = 3 μm, fibers with 500 nm diameter and 5 μm length, and 55% void without conductive agents and binders. **a**, 3D microstructure models with various particle/fiber ratios. **b**, Illustrations of percolation networks for the ion transport paths and average tortuosity values (τ).

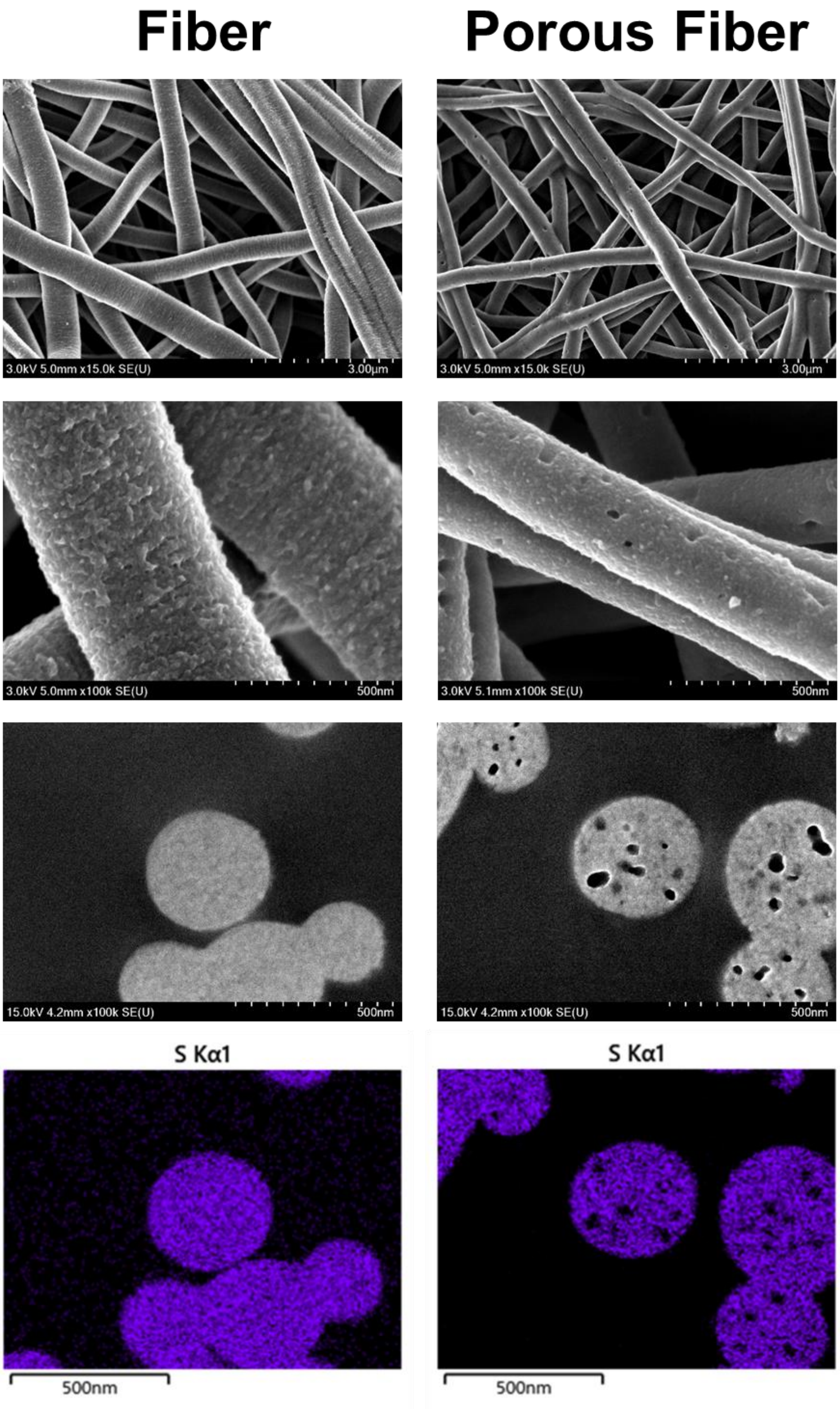

Supplementary Fig. 11 | SEM and EDX (sulfur mapping) images of SPAN fiber and porous fiber.

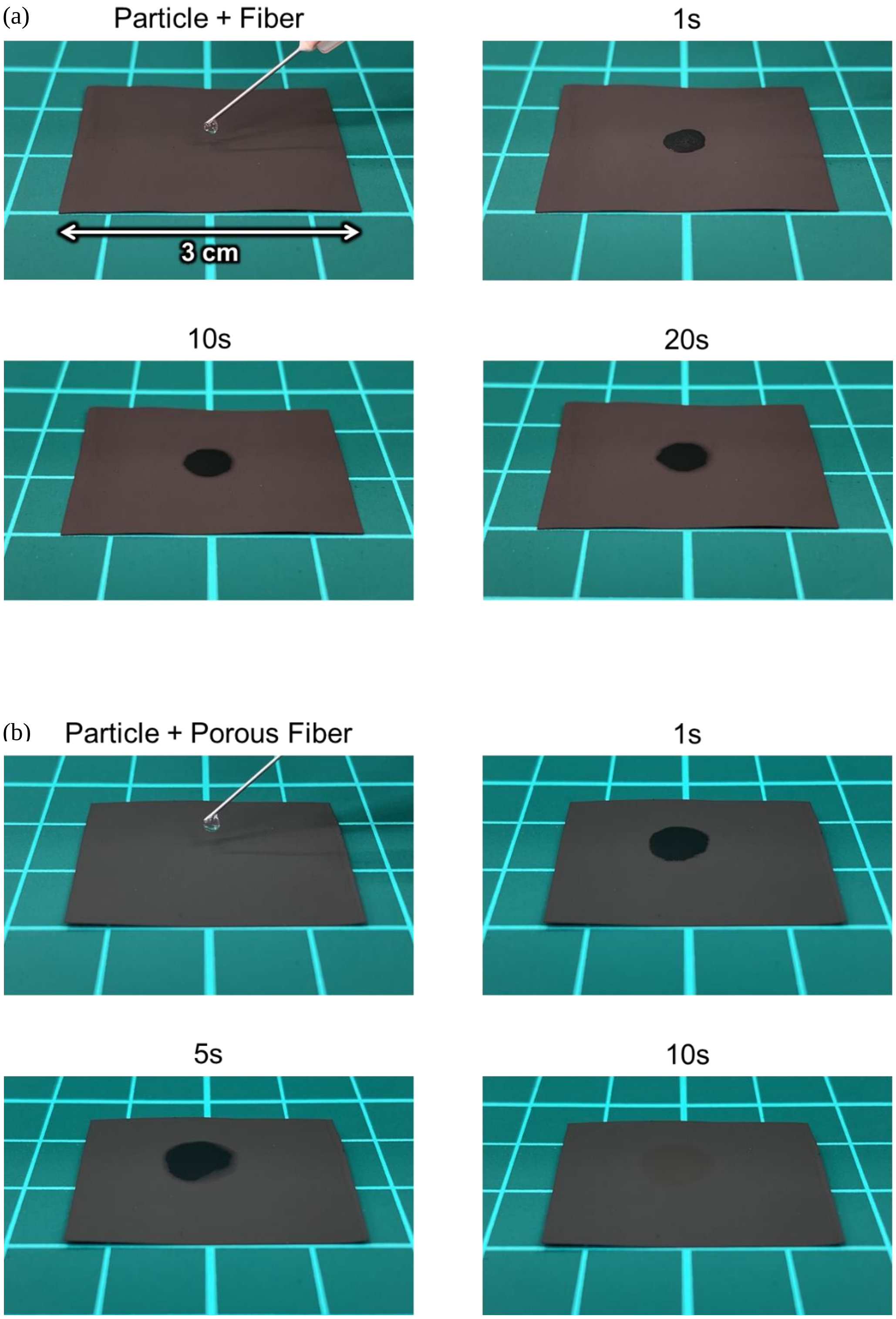

Supplementary Fig. 12 | Photographs of SPAN cathodes mixing SPAN particle and SPAN fibers when an electrolyte solution was dropped. **a**, SPAN non-porous fiber. **b**, SPAN porous fiber.

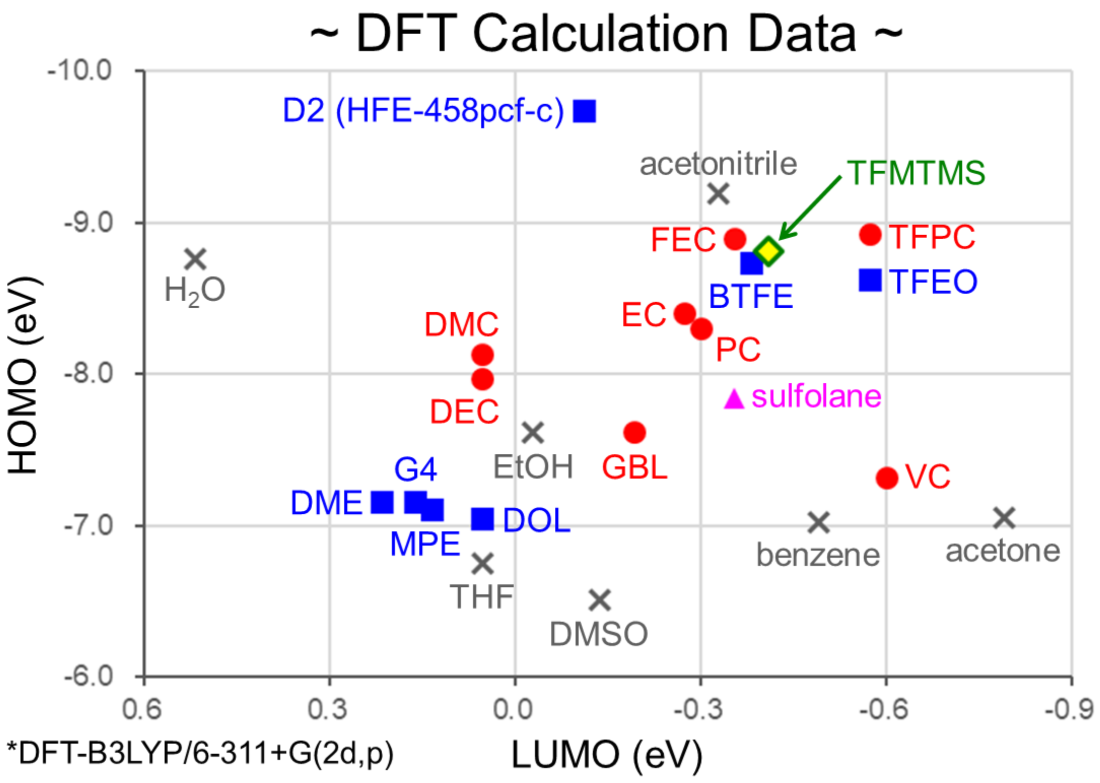

Supplementary Fig. 13 | Energy levels in frontier orbitals (HOMO and LUMO) of various solvents for the electrolyte solutions used in this work, calculated with the Gaussian 09 program package by DFT at the B3LYP/6-311+G(2d,p) level[6–8].

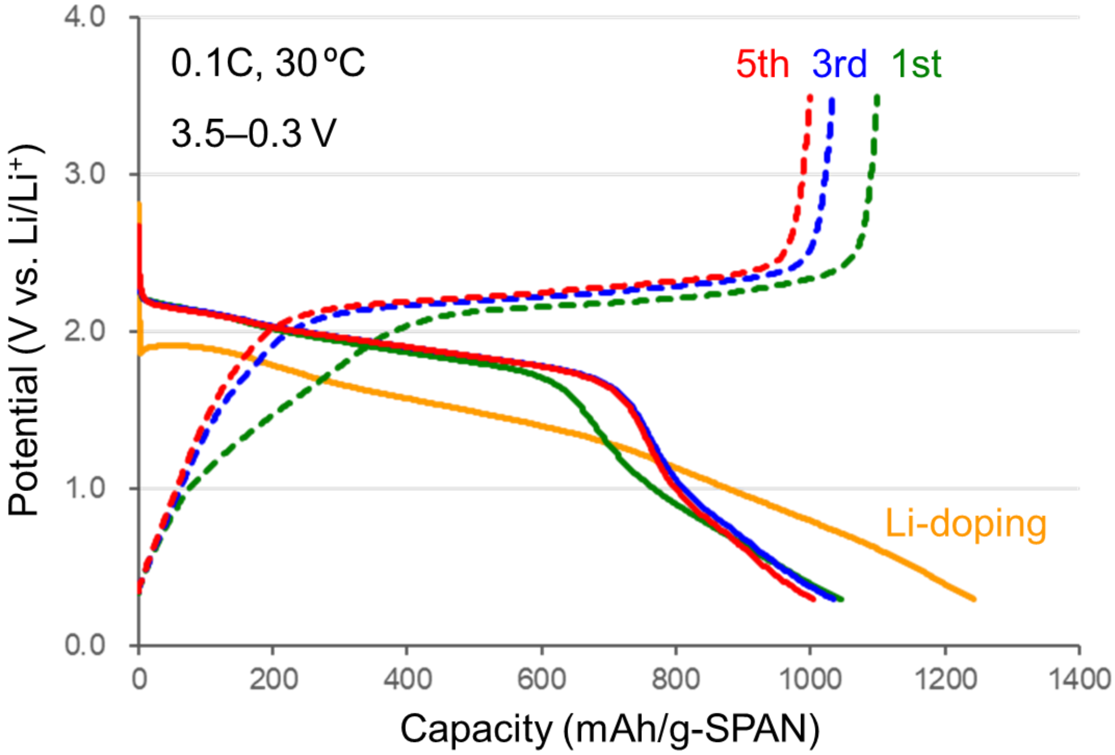

Supplementary Fig. 14 | Chg./dischg. characteristic curves for the electrochemical prelithiation with five cycles of the thick SPAN cathode in the 3.5-0.3 V and 0.1C-rate operation at 30 ºC.

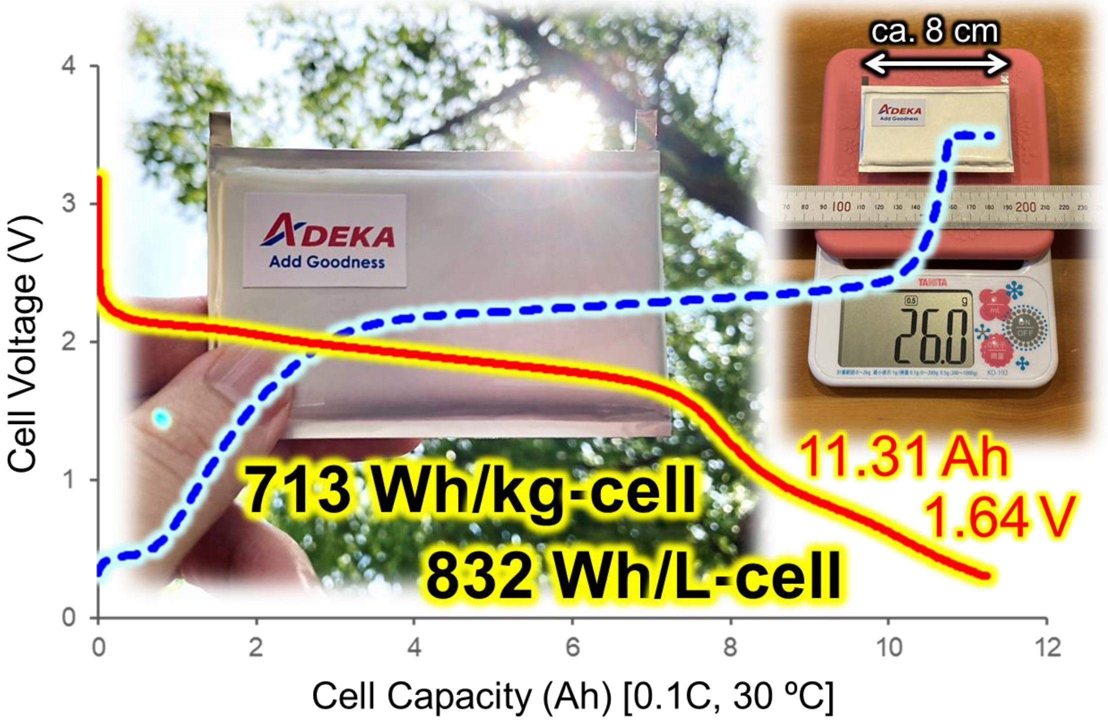

Supplementary Fig. 15 | Reversible chg./dischg. characteristic curves of the ultra-lightweight Li–SPAN pouch cell with the ALIS-PC design in the 3.5-0.3 V and 0.1C-rate operation at 30 ºC.

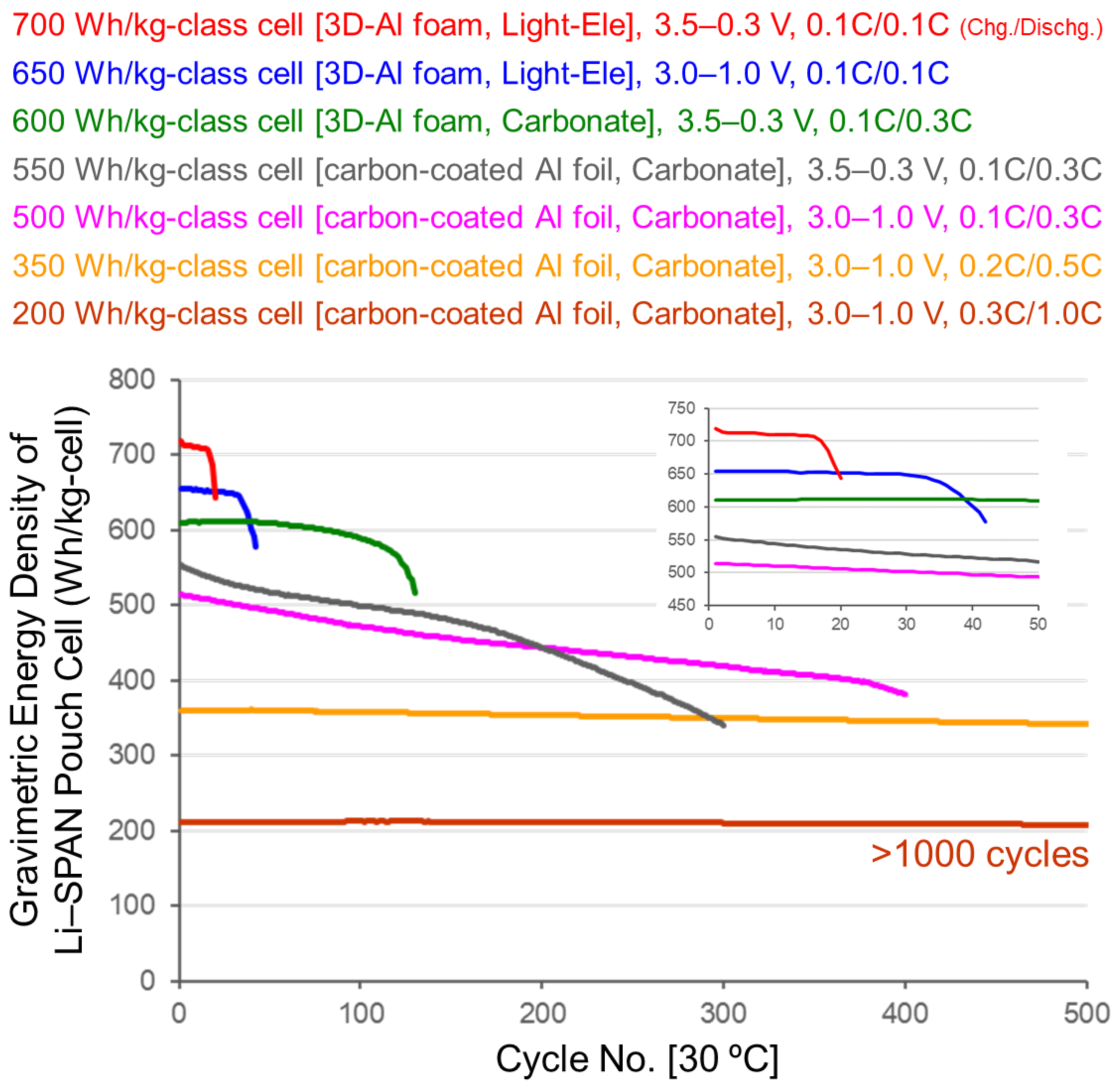

Supplementary Fig. 16 | Chg./dischg. cycle performances of Li–SPAN pouch cells with various energy density designs.

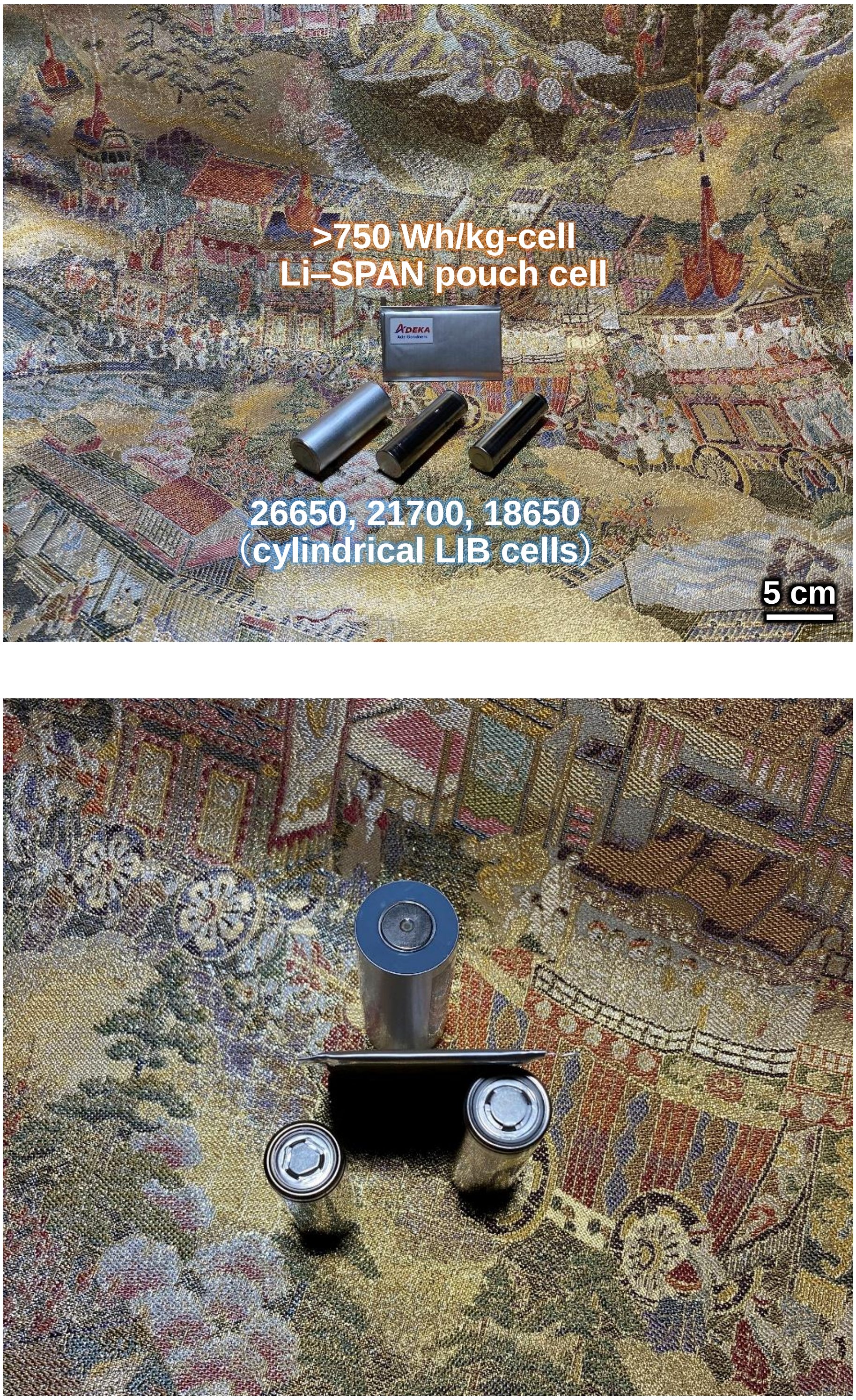

Supplementary Fig. 17 | Photographs of the ultra-lightweight Li–SPAN pouch cell (>750 Wh/kg-cell) fabricated in this work and commercial cylindrical LIB cells of 18650, 21700, and 26650 sizes placed on a Japanese kimono obi belt (weaved in Kiryu, Gunma, Japan).

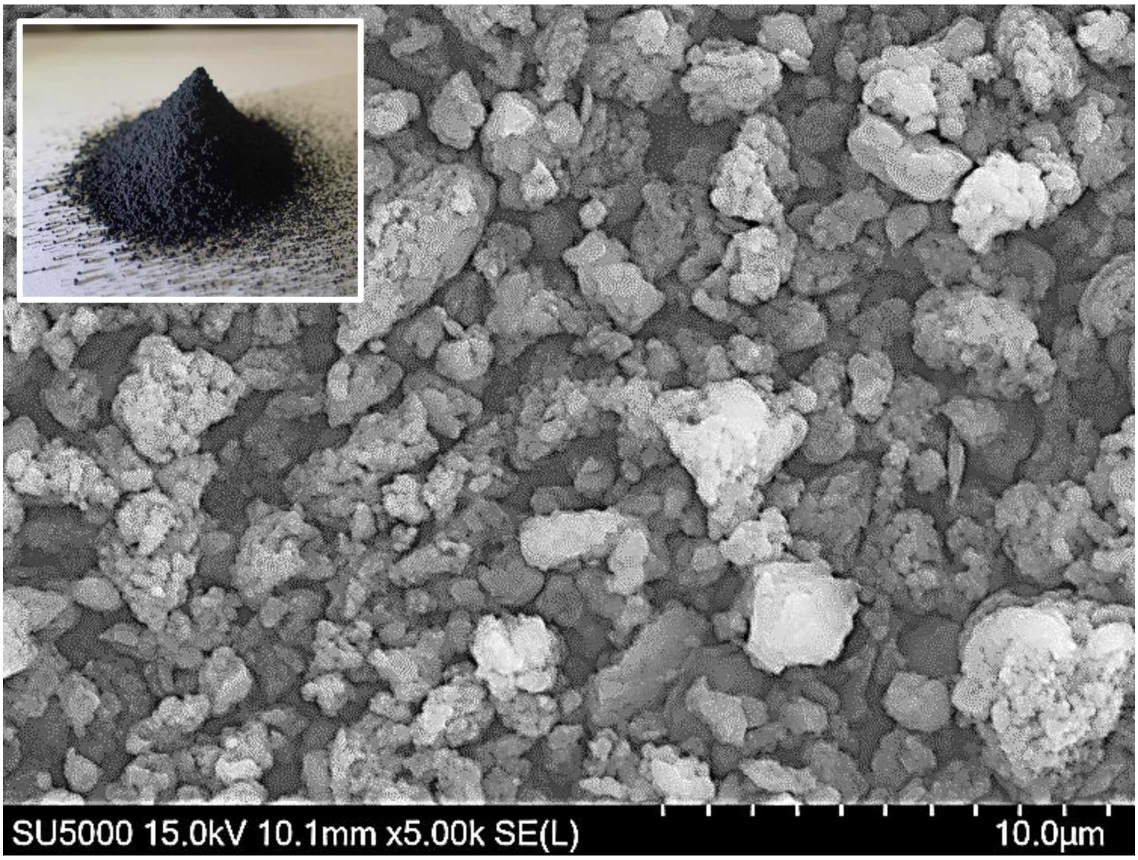

Supplementary Fig. 18 | Photograph and SEM image of the SPAN particle (ADEKA AMERANSA SAM-8).

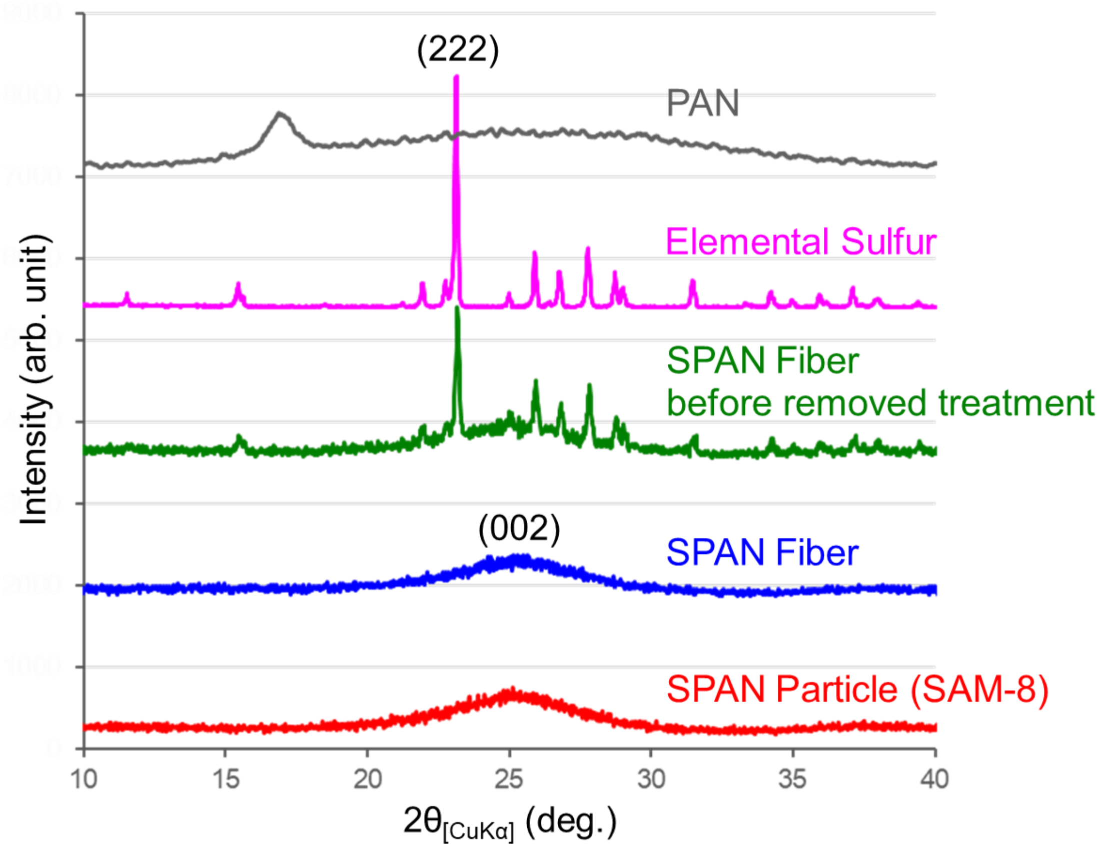

Supplementary Fig. 19 | Powder XRD analyses of various SPANs, elemental sulfur, and polyacrylonitrile (PAN).

Supplementary Table 1 | The highest and representative values in gravimetric energy densities of various rechargeable battery cells (presumptive densities are not included.)

Battery Type	Cell Energy Density [Wh/kg-cell] (C-rate)	Ref
LIB (product)	250–300	9–12
LIB (development)	300–350	13–15
LIB [Si anode] (development)	350–500	16,17
Li–S (research) *unstable (only first dischg.)	695 (0.005C)	18
Li–S (research)	300–600	19–27
Li–S (development)	609	26,27
Li–S (development)	300–600	26–31
**Li–S (SPAN / this work)**	**713 (0.1C)**	**—**
**Li–S (SPAN / this work)**	**761 (0.05C)**	**—**
LMB (research)	564 (0.1C)	32
LMB (development)	524 (0.1C)	33
LMB (research & development)	300–550	33–41
LAB (research)	543 (0.1C)	42

Supplementary Table 2 | Characteristics of various electrolyte solutions and densities of solid electrolytes for lithium-based rechargeable batteries

Electrolyte Solution	*d* (g/cm^3)	*η* [mPa·s]	*σ* [mS/cm]		Ref
1.0 M $LiPF_6$ in EC/EMC (30/70, vol%)	1.21	3.4	8.1	at 25 ºC	—
1.0 M $LiPF_6$ in EC/DEC (50/50, vol%)	1.25	—	—	at 25 ºC	—
1.0 M $LiPF_6$ in EC/DEC (1/2, vol)	1.21	3.8	10.6	at 30 ºC	43
1.0 M $LiPF_6$ in FEC/DEC (50/50, vol%)	1.32	—	—	at 25 ºC	—
LiFSA : DMC = 1 : 10 (molar ratio) *1.08 M	1.18	1.5	9.9	at 30 ºC	44
LiFSA : DMC = 1 : 2.9 (molar ratio) *3.04 M	1.36	12.9	8.1	at 30 ºC	44
LiFSA : DMC = 1 : 1.1 (molar ratio) *5.49 M	1.57	238.9	1.1	at 30 ºC	44
LiFSA : EC = 1 : 10 (molar ratio) *1.34 M	1.43	8.1	9.7	at 30 ºC	44
LiFSA : EC = 1 : 2.9 (molar ratio) *3.56 M	1.57	103.2	2.3	at 30 ºC	44
LiFSA : EC = 1 : 1.3 (molar ratio) *5.67 M	1.71	621.9	0.8	at 30 ºC	44
1.0 M LiTFSI in DOL/DME (50/50, vol%) + 2 wt% $LiNO_3$	1.17	1.7	12.3	at 25 ºC	45
1.0 M LiTFSA + 0.2 M $LiNO_3$ in DOL/DME (50/50, vol%)	1.14	1.6	13	at 30 ºC	46
7 m LiTFSI in DOL/DME (50/50, vol%)	1.52	ca. 150	1.0	at 25 ºC	45
[Li(G4)][TFSA]	1.40	106.0	1.6	at 30 ºC	47
[Li(G4)][TFSA]–4.0HFE	1.49	5.0	5.6	at 30 ºC	46
[Li(SL)$_2$][TFSA]–4.0HFE	1.55	8.7	0.7	at 30 ºC	46
0.2 m LITFSI + 0.4 m $LiNO_3$ in DME/MPE (48/52, vol%)	0.83	0.6	3.5	at 25 ºC	48
0.4 m LiTFSI + 0.4 m $LiNO_3$ + 0.1 M LiHFDF in DME/DOL/TFMTMS (48/17/35, vol%)	1.02	1.1	7.2	at 25 ºC	45
0.2 M LiTFSI + 0.2 M LiFSI + 0.1 M $LiNO_3$ + 0.1 M LiHFDF in DME/DOL/TFMTMS (75/5/20, vol%)	0.98	0.8	9.6	at 30 ºC	This Work

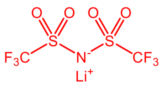
LiTFSI

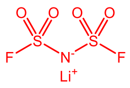
LiFSI

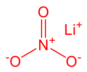
$LiNO_3$

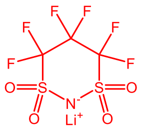
LiHFDF

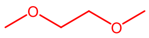
DME

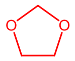
DOL

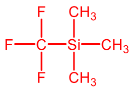
TFMTMS

Solid Electrolyte	*d* (g/cm^3)	Ref
$75Li_2S·25P_2S_5$ [LPS]	1.88	49
Li_6PS_5Cl [LPSCl]	1.64	50
$Li_{10}GeP_2S_{12}$ [LGPS]	ca. 2	51,52
$Li_7La_3Zr_2O_{12}$ [LLZO]	ca. 5	51–53
$Li_{1+x}Al_xTi_{2-x}(PO_4)_3$ [LATP]	ca. 3	51
PEO / LiTFSI	ca. 1.2	52
(PVDF-based polymer / electrolyte solution)	1.84	54
Complex hydride	ca. 1	55
$LiBH_4$, LiI (for complex hydride)	0.666, 3.49	56

Supplementary Table 3 | Correlation between separator properties and chg./dischg. short-circuit phenomenon

Separator	Thickness (μm)	Porosity (%)	Short-circuit phenomenon after 50 cycles
GA-100 (Glass Fiber)	440	40-80	stable
Non–woven (PAN)	50	>40	stable
Non–woven (PAN)	30	>40	micro-short
Non–woven (PAN)	10	>40	short
Celgard 2325 (PP/PE/PP)	25	39	stable
Celgard 2400 (PP)	25	41	stable
Microporous membrane (PP)	20	40	stable
Microporous membrane (PP)	17	39	micro-short
Microporous membrane (PE)	15	38	stable
Microporous membrane (PE)	10	37	short
SETELA-05C (PE)	5	35	stable

Supplementary Table 4 | A design sheet of the ultra-lightweight Li–SPAN pouch cell

**Ultra-lightweight pouch-type cell**											
A/S/C/S/A/S/C/S/A/S/C/S/A/S/C/S/A/S/C/S/A											
*A : Anode (Li-metal), C : Cathode (SPAN), S : Separator											
**Cathode (A)**											
3D-Al foam	8.50×4.20=	35.7	cm2	6.90	mg/cm2	0.246	g/sheet	5	sheets	1.23	g
**porous 3D-Al foam (Al-CELMET) sheet weight-saved by the laser-drilling technique*											
SPAN layer	8.00×4.20=	33.6	cm2								
	porosity	0.36									
	density	1.2	g/cm3								
	thickness	578	μm					5	sheets	2.89	mm
	thickness		**after Lithiation*		**coin cell data : 3.5-0.3 V ⇒ 0.3 V, 0.1C-rate, 30 ℃*					4.15	mm
	SPAN loading	68.0	mg-SPAN/cm2								
	**Sulfur loading : 32.6 mg-S/cm2*										
		SPAN	0.980	wt. ratio	1.9	g/cm3					
		SWCNT	0.0040	wt. ratio	2.1	g/cm3					
		SBR	0.0070	wt. ratio	0.94	g/cm3					
		CMC,CNF	0.0090	wt. ratio	1.59	g/cm3					
	Weight	69.4	mg/cm2			2.33	g/sheet	5	sheets	11.7	g
	Weight		**after Lithiation*		**coin cell data : 3.5-0.3 V ⇒ 0.3 V, 0.1C-rate, 30 ℃*					15.6	g
	Capacity	1000	mAh/g-SPAN	68.0	mAh/cm2	2.28	Ah/sheet	5	sheets	11.4	Ah
	**Reversible Capacity (3.5-0.3V, 0.1C-rate, 30 ℃)*										
**Anode (B)**											
Li-metal	8.70×4.40=	38.3	cm2								
**without Cu foil*											
	thickness	10	μm/sheet					6	sheets	0.060	mm
	porosity	0									
	density (Li)	0.53	g/cm3								
	Li loading	0.53	mg/cm2								
	Weight	0.53	mg/cm2			0.020	g/sheet	6	sheets	0.12	g
**Separator (C)**											
SETELA-05C	8.40×4.60=	72.2	cm2								
	thickness	5	μm/sheet					10	sheets	0.050	mm
	porosity	0.35									
	density (PE)	0.95	g/cm3								
	Weight	0.31	mg/cm2			0.022	g/sheet	10	sheets	0.22	g
**Electrolyte (D)**											
Light-Ele	density	0.98	g/cm3								
	Weight	7.27	g		*E/S(μL/mg-S)		1.35				
**Al-laminated film & Tab (E)**											
DNP Battery Pouch	9.00×5.50=	49.5	cm2								
	thickness	77	μm/sheet					2	sheets	0.154	mm
	Weight	0.013	g/cm2			0.644	g/sheet	2	sheets	1.29	g
Al tab	Weight	0.08	g	**4 mm×7.5 cm×t=0.1 mm*							
Ni tab	Weight	0.26	g	**4 mm×7.5 cm×t=0.1 mm*							
**Cell**											
Capacity (Dischg.)	11.4	Ah									
Voltage (Dischg.;Av.)	1.64	V									
Energy (Dischg.)	18.7	Wh									
Weight (Content:A+B+C+D)	24.4	g									
Energy Density (Content)	766	Wh/kg	**excluding weights of pouch and tabs*								
Weight (Cell:A+B+C+D+E)	26.1	g									
Energy Density (Cell)	719	Wh/kg	**based on total mass of all cell components*								
Thickness (Cell)	4.41	mm									
Size (Cell)	49.5	cm2									
Volume (Cell)	21.8	cm3									
Energy Density (Cell)	857	Wh/L	**based on total mass of all cell components*								

Weight distribution

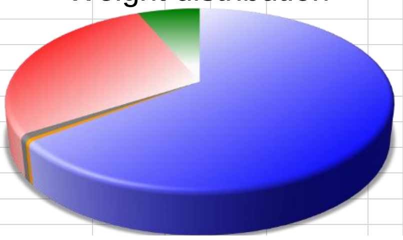

Cathode : 64.56% Anode : 0.46%
Separator : 0.84% Electrolyte : 27.89%
Pouch & Tab : 6.25%

Supplementary References

1. Ahmed, M. S., Lee, S., Agostini, M., Jeong, M.-G., Jung, H.-G., Ming, J., Sun, Y.-K., Kim, J., & Hwang, J.-Y. Multiscale understanding of covalently fixed sulfur–polyacrylonitrile composite as advanced cathode for metal–sulfur batteries. *Adv. Sci.* **8**, 2101123 (2021).
2. Zhao, X., Wang, C., Li, Z., Hu, X., Razzaq, A. A. & Deng, Z. Sulfurized polyacrylonitrile for high-performance lithium sulfur batteries: advances and prospects. *J. Mater. Chem. A* **9**, 19282–19297 (2021).
3. Zhang, S. S. Understanding of sulfurized polyacrylonitrile for superior performance lithium/sulfur battery. *Energies* **7**, 4588–4600 (2014).
4. Takemoto, K., Wakasugi, J., Kubota, M., Kanamura, K. & Abe, H. Dual additive of lithium titanate and sulfurized pyrolyzed polyacrylonitrile in sulfur cathode for high rate performance in lithium–sulfur battery. *Phys. Chem. Chem. Phys.* **25**, 351–358 (2023).
5. Wang, F. & He, X. A multi-functional sulfurized polyacrylonitrile interlayer for lithium sulfur batteries. *Mater. Lett.* **256**, 126604 (2019).
6. Gaussian 09, Revision D.01, Frisch, M. J. et al. Gaussian, Inc., Wallingford CT (2009).
7. Lee, C. Yang, W. & Parr, R. G. Development of the Colle-Salvetti correlation-energy formula into a functional of the electron density. *Phys. Rev. B* **37** 785 (1988).
8. Becke, A. D. Density-functional thermochemistry. III. The role of exact exchange. *J. Chem. Phys.* **98**, 5648–5652 (1993).
9. Edström, K. et al. *BATTERY 2030+ Roadmap* Fig. 9 https://battery2030.eu/wp-content/uploads/2023/09/B-2030-Science-Innovation-Roadmap-updated-August-2023.pdf (2023).
10. Houache, M. S. E., Yim, C.-H., Karkar Z. & Abu-Lebdeh, Y. On the current and future outlook of battery chemistries for electric vehicles—mini review. *Batteries* **8**, 70 (2022).
11. Liu, W., Placke, T. & Chau, K.T. Overview of batteries and battery management for electric vehicles. *Energy Reports* **8**, 4058–4084 (2022).
12. König, A., Nicoletti, L., Schröder, D., Wolff, S., Waclaw, A. & Lienkamp, M. An Overview of parameter and cost for battery electric vehicles. *World Electr. Veh. J.* **12**, 21 (2021).
13. Contemporary Amperex Technology Co., Ltd. *CATL, R&D Innovative Technology (High Energy Density Technology Exceeding Limitations Battery cell energy density: 330 Wh/kg)* https://www.catl.com/en/research/technology/ (2022–2023).
14. Wang, L., Wang, J., Wang, L., Zhang, M., Wang, R. & Zhan, C. A critical review on nickel-based cathodes in rechargeable batteries. *Int. J. Miner. Metall. Mater.* **29**, 925–941 (2022).
15. Cano, Z. P., Banham, D., Ye, S., Hintennach, A., Lu, J., Fowler, M. & Chen, Z. Batteries and fuel cells for emerging electric vehicle markets. *Nat. Energy* **3**, 279–289 (2018).

16. GS Yuasa Corp. *News Release (Improvement to Silicon Based Negative Electrodes Makes Major Progress in Practical Use of Advanced Batteries -Development of technology that achieves both high energy density and lifetime performance-)* https://newsroom.gs-yuasa.com/en/news-release/113 (2023).
17. Amprius Technologies, Inc. *RECENT ANNOUNCEMENTS (The All-New Amprius 500 Wh/kg Battery Platform is Here)* https://amprius.com/the-all-new-amprius-500-wh-kg-battery-platform-is-here/ (2023).
18. Cheng, Q., Chen, Z.-X., Li, X.-Y., Hou, L.-P., Bi, C.-X., Zhang, X.-Q., Huang, J.-Q. & Li, B.-Q. Constructing a 700 Wh kg^{-1}-level rechargeable lithium–sulfur pouch cell. *J. Energy Chem.* **76**, 181–186 (2023).
19. Ye, G., Zhao, M., Hou, L.-P., Chen, W.-J., Zhang, X.-Q., Li, B.-Q. & Huang, J.-Q. Evaluation on a 400 Wh kg^{-1} lithium–sulfur pouch cell. *J. Energy Chem.* **66**, 24–29 (2022).
20. Li, S., Ishikawa, S., Liu, J., Ueno, K., Dokko, K., Inoue, G. & Watanabe, M. Importance of mass transport in high energy density lithium–sulfur batteries under lean electrolyte conditions. *Batteries Supercaps* **5**, e202100409 (2022).
21. Yoshie, Y., Hori, K., Mae, T. & Noda, S. High-energy-density Li–S battery with positive electrode of lithium polysulfides held by carbon nanotube sponge. *Carbon* **182**, 32–41 (2021).
22. Wird, M. & Offer, G. J. (Eds.) *Lithium–Sulfur Batteries* (Wiley, 2019).
23. Chen, J., Henderson, W. A., Pan, H., Perdue, B. R., Cao, R., Hu, J. Z., Wan, C., Han, K. S., Mueller, K. T., Zhang, J.-G., Shao, Y. & Liu, J. Improving lithium–sulfur battery performance under lean electrolyte through nanoscale confinement in soft swellable gels. *Nano Lett.* **17**, 3061–3067 (2017).
24. Ye, Y., Wu, F., Liu, Y., Zhao, T., Qian, J., Xing, Y., Li, W., Huang, J., Li, L., Huang, Q., Bai, X. & Chen, R. Toward practical high-energy batteries: a modular-assembled oval-like carbon microstructure for thick sulfur electrodes. *Adv. Mater.* **29**, 1700598 (2017).
25. Demir-cakan, R. (Ed.) *Li-S Batteries: The Challenges, Chemistry, Materials and Future Perspectives* (World Scientific Publishing, 2017).
26. Zhou, G., Chen, H. & Cui, Y. Formulating energy density for designing practical lithium–sulfur batteries. *Nat. Energy* **7**, 312–319 (2022).
27. Tan, J., Yao, Z., Ye, M. & Shen, J. An optimized combination inspired by the wooden-barrel effect for Li–S pouch cells. *Cell Rep. Phys. Sci.* **2**, 100659 (2021).
28. LG Energy Solution Ltd. *Company News / SUPPLEMENTARY STORIES (LG Energy Solution Shines at InterBattery 2023)* https://news.lgensol.com/company-news/supplementary-stories/1651/ (2023).
29. Lyten, Inc. *Products / Batteries (LYTCELL™ LITHIUM-SULFUR BATTERIES)* https://lyten.com/products/batteries/ (2022–2023).

30. GS Yuasa Corp. *News Release (GS Yuasa Achieves R&D Milestone in NEDO Advanced Aircraft System Commercialization Project –Successful demonstration of 400Wh/kg-class lithium-sulfur battery–)* https://www.gs-yuasa.com/en/newsrelease/article.php?ucode=gs211115465619_1089 (2021).
31. Dörfler, S., Walus, S., Locke, J., Fotouhi, A., Auger, D. J., Shateri, N., Abendroth, T., Härtel, P., Althues, H. & Kaskel, S. Recent progress and emerging application areas for lithium–sulfur battery technology. *Energy Technol.* **9**, 2000694 (2021).
32. Wu, F., Fang, S., Kuenzel, M., Mullaliu, A., Kim, J.-K., Gao, X., Diemant, T., Kim, G.-T. & Passerini, S. Dual-anion ionic liquid electrolyte enables stable Ni-rich cathodes in lithium-metal batteries. *Joule* **5**, 2177–2194 (2021).
33. Enpower Greentech Inc. & Enpower Japan Corp. *Press Release (Enpower Successfully Develops a Record-Breaking Lithium Metal Battery)* https://enpowerjp.co.jp/wp-content/uploads/2021/10/Enpower-News-Release-2021-10-25_English.pdf (2021).
34. SoftBank Corp. & HAPSMobile Inc. *Press Release (SoftBank Corp. Develops Battery Pack with Next-generation Lithium-metal Battery Cells and Successfully Demonstrates Operation in the Stratosphere)* https://www.softbank.jp/en/corp/news/press/sbkk/2023/20230316_01/ (2023).
35. Cuberg, *Latest news (Cuberg aviation battery systems announcement earns widespread media attention)* https://cuberg.net/news/cuberg-aviation-battery-systems-announcement-earns-widespread-media-attention (2023).
36. Enpower Greentech Inc. *Press Release (Enpower Greentech Achieved Breakthrough in Cylindrical Batteries)* https://enpowerjp.co.jp/wp-content/uploads/2022/11/JP_Enpower-News-Release_English_20221104.pdf (2022).
37. Enpower Technology Co. & Enpower Greentech Inc. & Enpower Japan Corp. *Press Release (Enpower Greentech develops world's lightest 100 Ah lithium metal battery)* https://enpowerjp.co.jp/wp-content/uploads/2022/07/EN_Enpower-News-Release_20220710_ PDF.pdf (2022).
38. SES AI Corp. *Cell Data (SES 50Ah Li-Metal Cell Data Report)* https://ses.ai/wp-content/uploads/2023/02/SES-50Ah-Li-Metal-Cell-Data-Report.pdf (2022).
39. Sion Power Corp. *NEWS (Sion Power Demonstrates More Than 2500 Cycles in Licerion® Rechargeable Battery Technology)* https://sionpower.com/2022/sion-power-demonstrates-more-than-2500-cycles-in-licerion-rechargeable-battery-technology/ (2022).
40. Deng, W., Dai, W., Zhou, X., Han, Q., Fang, W., Dong, N., He, B. & Liu, Z. Competitive solvation-induced concurrent protection on the anode and cathode toward a 400 Wh kg^{-1} lithium metal battery. *ACS Energy Lett.* **6**, 115–123 (2021).
41. Niu, C., Lee, H., Chen, S., Li, Q., Du, J., Xu, W., Zhang, J.-G., Whittingham, M. S., Xiao, J., & Liu, J. High-energy lithium metal pouch cells with limited anode swelling and long stable cycles. *Nat. Energy* **4**, 551–559 (2019).

42. Matsuda, S., Ono, M., Yamaguch, S. & Uosaki, K. Criteria for evaluating lithium–air batteries in academia to correctly predict their practical performance in industry. *Mater. Horiz.* **9**, 856–863 (2022).
43. Takeyoshi, J., Kobori, N. & Kanamura, K. Electrochemical evaluation of lithium–metal anode in highly concentrated ethylene carbonate based electrolytes. *Electrochemistry*, **88**, 540–547 (2020).
44. Wang, J., Yamada, Y., Sodeyama, K., Chiang, C. H., Tateyama, Y. & Yamada, A. Superconcen-trated electrolytes for a high-voltage lithium-ion battery. *Nat. Commun.* **7**, 12032 (2016).
45. Liu, T., Shi, Z., Li, H., Xue, W., Liu, S., Yue, J., Mao, M., Hu, Y., Li, H., Huang, X., Chen, L. & Suo, L. Low-density fluorinated silane solvent enhancing deep cycle lithium–sulfur batteries' lifetime. *Adv. Mater.* **33**, 2102034 (2021).
46. Yanagi, M., Ueno, K., Ando, A., Li, S., Matsumae, Y., Liu, J., Dokko, K. & Watanabe, M. Effects of polysulfide solubility and Li ion transport on performance of Li–S batteries using sparingly solvating electrolytes. *J. Electrochem. Soc.* **167**, 070531 (2020).
47. Watanabe, M., Dokko, K., Ueno, K. & Thomas, M. L. From ionic liquids to solvate ionic liquids: challenges and opportunities for next generation battery electrolytes. *Bull. Chem. Soc. Jpn.* **91**, 1660–1682 (2018).
48. Liu, T., Li, H., Yue, J., Feng, J., Mao, M., Zhu, X., Hu, Y., Li, H., Huang, X., Chen, L. & Suo, L. Ultralight electrolyte for high-energy lithium–sulfur pouch cells. *Angew. Chem. Int. Ed.* **60**, 17547–17555 (2021).
49. Sakuda, A., Hayashi, A & Tatsumisago, M. Sulfide solid electrolyte with favorable mechanical property for all-solid-state lithium battery. *Sci. Rep.* **3**, 2261 (2013).
50. Lee, J. M., Park, Y. S., Moon, J.-W. & Hwang, H. Ionic and electronic conductivities of lithium argyrodite Li_6PS_5Cl electrolytes prepared via wet milling and post-annealing. *Front. Chem.* **9**, 778057 (2021).
51. Wu, B., Wang, S., Evans IV, W. J., Deng, D. Z., Yang, J. & Xiao, J. Interfacial behaviours between lithium ion conductors and electrode materials in various battery systems. *J. Mater. Chem. A* **4**, 15266–15280 (2016).
52. Zhao, Q. Interphases of polymer electrolytes. *Joule* **3**, 1569–1571 (2019).
53. Sakuda, A., Hayashi, A., Takigawa, Y., Higashi, K. & Tatsumisago, M. Evaluation of elastic modulus of $Li_2S–P_2S_5$ glassy solid electrolyte by ultrasonic sound velocity measurement and compression test. *J. Ceram. Soc. Jpn.* **121**, 946–949 (2013).
54. Nasef, M. M., Suppiah, R. R. & Dahlan, K. Z. M. Preparation of polymer electrolyte membranes for lithium batteries by radiation-induced graft copolymerization. *Solid State Ionics* **171**, 243–249 (2004).

55. Unemoto, A., Nogami, G., Tazawa, M., Taniguchi, M. & Orimo, S. Development of 4V-class bulk-type all-solid-state lithium rechargeable batteries by a combined use of complex hydride and sulfide electrolytes for room temperature operation. *Mater. Trans.* **58**, 1063–1068 (2017).
56. Merck KGaA, Products properties.
